# Modulation of gut microbiota and fecal metabolites by corn silk among high-fat diet-induced hypercholesterolemia mice

**DOI:** 10.3389/fnut.2022.935612

**Published:** 2022-08-01

**Authors:** Lin Ding, Shan Ren, Yaoxin Song, Chuangang Zang, Yuchao Liu, Hao Guo, Wenqing Yang, Hong Guan, Jicheng Liu

**Affiliations:** ^1^Department of Scientific Research, Science and Technology Achievement Transformation Center, Qiqihar Medical University, Qiqihar, China; ^2^College of Basic Medical, Qiqihar Medical University, Qiqihar, China; ^3^Qiqihar Academy of Medical Sciences, Qiqihar, China

**Keywords:** corn silk, high-fat diet, hypercholesterolemia, gut microbiota, fecal metabolites, bile acids

## Abstract

Corn silk (CS) is known to reduce cholesterol levels, but its underlying mechanisms remain elusive concerning the gut microbiota and metabolites. The aim of our work was to explore how altered gut microbiota composition and metabolite profile are influenced by CS intervention in mice using integrated 16S ribosomal RNA (rRNA) sequencing and an untargeted metabolomics methodology. The C57BL/6J mice were fed a normal control diet, a high-fat diet (HFD), and HFD supplemented with the aqueous extract of CS (80 mg/mL) for 8 weeks. HFD-induced chronic inflammation damage is alleviated by CS extract intervention and also resulted in a reduction in body weight, daily energy intake as well as serum and hepatic total cholesterol (TC) levels. In addition, CS extract altered gut microbial composition and regulated specific genera viz. *Allobaculum*, *Turicibacter*, *Romboutsia*, *Streptococcus*, *Sporobacter*, *Christensenella*, *ClostridiumXVIII*, and *Rikenella*. Using Spearman’s correlation analysis, we determined that *Turicibacter* and *Rikenella* were negatively correlated with hypercholesterolemia-related parameters. Fecal metabolomics analysis revealed that CS extract influences multiple metabolic pathways like histidine metabolism-related metabolites (urocanic acid, methylimidazole acetaldehyde, and methiodimethylimidazoleacetic acid), sphingolipid metabolism-related metabolites (sphinganine, 3-dehydrosphinganine, sphingosine), and some bile acids biosynthesis-related metabolites including chenodeoxycholic acid (CDCA), lithocholic acid (LCA), ursodeoxycholic acid (UDCA), and glycoursodeoxycholic acid (GUDCA). As a whole, the present study indicates that the modifications in the gut microbiota and subsequent host bile acid metabolism may be a potential mechanism for the antihypercholesterolemic effects of CS extract.

## Introduction

Recently there is a significant increase in the prevalence of hypercholesterolemia which turned out to be one of the most common pathological conditions among humans, and is characterized by elevated plasma/serum levels for total cholesterol (TC) and/or triglycerides (TG) as well as usually accompanied by abnormal levels of low-density lipoprotein cholesterol (LDL-C) and high-density lipoprotein cholesterol (HDL-C) (1). Many factors contribute to it, including congenital genetics, environment, and unhealthy eating habits. In the long run, hypercholesterolemia may be linked to chronic diseases such as atherosclerosis, coronary heart disease, and non-alcoholic fatty liver disease. Due to this, most current treatment strategies for hypercholesterolemia include a combination of lipid-lowering drugs along with lifestyle changes particularly low-fat as well as low-carbohydrate diets aimed at reducing plasma lipid levels with LDL-C as the main target (2). Thus, the development of natural herbs for improving and preventing hypercholesterolemia has become a hotspot for current research.

Researchers have shown that High-fat diets (HFD) alter the gut microbiota (GM) and act as a key influencer in the advancement of conditions like obesity, hyperlipidemia, resistance to insulin, and other diseases associated with metabolic syndromes (3, 4). There have been reports on the fecal microbiome and corresponding metabolites contributing to dyslipidemia in hosts (5, 6). By altering intestinal barrier permeability and interfering with reverse cholesterol transport certain bacteria and their metabolites like short-chain fatty acids (SCFAs) or bile acids (BAs) alters the efficiency of energy harvesting as well as susceptibility toward metabolic dysfunctions including obesity, diabetes, and non-alcoholic fatty liver disease (7–11). With the in-depth study of GM, more and more studies have confirmed the close relationship between GM and the therapeutic activity of the Chinese herbal medicines. Oral herbal medicines are often not directly absorbed by the host (e.g., polysaccharides) and instead enter the intestine where they are converted into active metabolites by the GM to exert their therapeutic effects (12).

The traditional herbs corn silk (Maydis stigma, CS) has been considered a folk medicine for centuries in countries such as China, Korea, Vietnam, the United States, and many more countries for health benefits to provide relief from conditions such as inflammation, edema, hyperlipidemia, hyperglycemia, hypertension, and obesity (13–16). The aqueous extract of CS is found to contain polysaccharides, alkaloids, flavonoids, phenols, saponins, tannins, and phytosterols (17). The Chinese Ministry of Health 2012 classified CS as a general food based on its long history of consumption, and no safety issues were found to exist. Research in recent years has reported that CS extract can significantly decrease the levels of serum TC, TG, and LDL-C in animal models, and is effective in decreasing the regulatory pool of hepatic cholesterol, in line with decreased blood and hepatic levels of cholesterol through modulation of mRNA expression levels of 3-hydroxy-3-methyl glutaryl- coenzyme A reductase (HMG-CoA reductase), cholesterol acyltransferase (ACAT), and farnesoid X receptor (FXR) (18, 19). In a study on the GM of mice with type-2 diabetes, it was found that after 5 weeks of treatment with CS polysaccharide, the blood glucose of diabetic mice was significantly reduced and the quality and quantity of *Lactobacillus* and *Bacteroides* genus in the feces of mice increased significantly, and CS polysaccharide was considered to have prebiotic properties (20).

Hence, the main focus of this work is to evaluate the ameliorative impacts of CS extract on HFD-induced hypercholesterolemia among mice, as well as its effect on the composition of microbial communities and metabolites in feces. This report provides valuable insight into the underlying mechanism through which CS exerts its effect on hypercholesterolemia.

## Materials and methods

### Materials and reagents

We used a control diet containing 10, 20, and 70% of lipids, proteins, and carbohydrates, respectively, whereas the HFD contains 40.29% lipids, 17.71% proteins, and 42% carbohydrates (Beijing HFK Bioscience Co., Ltd., China). The HFD consisted of lard 10%, sucrose 15%, egg yolk powder 15%, casein 5%, cholesterol 1.2%, sodium cholate 0.2%, calcium hydrogen phosphate 0.6%, rock flour 0.4%, and rat maintenance diet 52.6%, the energy values were 3.85 kcal/g and 4.40 kcal/g for the control and HFD, respectively. The CS extract was obtained from Science and Technology Achievement Transformation Center (Qiqihar Medical University, China). For the preparation of CS extract, the CS was washed with ultrapure water, dried at room temperature, and then approximately 20 kg of dried CS was extracted with boiling water (1:15 w/v) using a vacuum reflux extraction and concentration unit (Chengdong Medicine Machine Instrument Factory, Wenzhou, China) for at least 60 min each time, three times in total. After this, the concentrated solution is spray-dried (GEA, Dusseldorf, Germany) to obtain CS extract. Meanwhile, the yield of crude extract was 4.74% which is subjected to total glucose estimation by phenol-sulfuric acid method, protein determination by Kjeldahl method, total flavonoids analysis by AlCl3 spectrophotometric method, and total phenols measurement using forintanol method. CS extract had a total glucose content of 34.42%, polysaccharide content of 16.63%, protein content of 26.34%, a total flavonoid content of 4.07%, and a total phenolic content of 10.17%.

### Animals and experimental design

All animal experiments were conducted as per national legislation and local guidelines at the Center for Laboratory Animals, Qiqihar Medical University, Qiqihar, China. We purchased male C57BL-6J mice weighing 16–20 g from Changchun Changsheng Experimental Animal Co., Ltd., (Changchun, China). The maintenance of mice was achieved in an environment [i.e., specific-pathogen-free (SPF), 12 h light/dark cycles, a temperature of 20–22°C, and 45 ± 5% humidity]. To acclimate the mice, they were placed on a control diet, administered *ad libitum* for 7 days and divided randomly into groups of three with eight mice each: Control group mice received a control diet with ultrapure water (10 mL/kg), HFD group mice received a HFD with ultrapure water (10 mL/kg), and CS group mice received HFD with an aqueous solution of CS extract (80 mg/mL). CS group was administered with 800 mg/kg per day dosage. For 8 weeks, all mice were made freely accessible to control diet/HFD as well as water, and their weights along with food intake consumption were recorded once every week. The Animal Ethical Care Committee of Qiqihar Medical University reviewed and approved all animal experimental procedures (approval number QMU-AECC-2020-69). After fasting overnight, mice were euthanized on concluding the experiments. The feces were collected for subsequent analysis, and collected blood samples were kept for 30 min at room temperature to guarantee thorough clotting, then centrifuged at 3,500 rpm for 20 min to extract the serum samples. We measured wet weights of liver, epididymal fat, and perinephric fat tissues while dividing them into sections of two where one of the sections was fixed using a formalin solution (10%) for histological examination, and the other was frozen instantly using liquid nitrogen and then placed in a freezer (−80°C) till further utilization.

### Glucose Tolerance Test

Mice were fasted overnight and orally gavaged with glucose in sterile water (2 g/kg). Then, blood samples were collected from the tip of the tail vein at 0, 30, 60, and 120 min after glucose administration, and blood glucose was measured with a blood glucose meter (Roche Diagnostics GmbH, Mannheim, Germany).

### Biochemistry assays

An auto-biochemistry analyzer (Olympus AU 600, Hamburg, Germany) was used to measure the levers of TC, TG, LDL cholesterol (LDL-C), HDL cholesterol (HDL-C), and enzymes like alanine transaminase (ALT), and aspartate transaminase (AST). We also quantified the hepatic TC and TG levels using commercial kits (Nanjing Jian Cheng Biology Engineering Institute, Nanjing, China). The levels of IL-6 and TNF-α in serum and hepatic were assessed using ELISA kits (Shanghai Jianglai Biotechnology, Shanghai, China).

### Histopathological analysis

The histology analysis was carried out as described in the previous report (21). For this, the liver and adipose tissue samples were fixed by the addition of 4% paraformaldehyde solution and later embedded in paraffin. A three-micron section was cut and subjected to staining with hematoxylin and eosin. The histological changes are being observed using a light microscope (CX31, Olympus, Tokyo, Japan).

### Gut microbiota analysis

Following the manufacturer’s instructions, microbial DNA was extracted from fecal samples with a DNA extraction kit (Qiagen, Germany). The regions V3–V4 in the bacterial 16S ribosomal-RNA genes were amplified through PCR analysis using the following primers: forward primer (341F5′-CCTACGGGRSGCAGCAG-3′) and reverse primer (806R5′-GGACTACVVGGGTATCTAATC-3′). Following the manufacturer’s guidelines, the AxyPrep DNA Gel Extraction Kit (Axygen Biosciences, Union City, CA, United States) has been utilized for the amplicons purification after being extracted from 2% agarose gels, followed by quantification with the aid of Qubit 2.0 (Invitrogen, United States). Following library preparation, these tags have been sequenced using a MiSeq platform (from Illumina, Inc., CA, United States). The construction of the library, as well as sequencing, were carried out at Realbio Genomics Institute (Shanghai, China). UPARSE^1^ was applied with a similarity cut-off value of 97% for clustering the Operational Taxonomic Units (OTUs) while Usearch (version 7.0) for identification and removal of chimeric sequences. RDP Classifier^2^ assisted in assigning representative tags to taxa based on the RDP database (see text footnote 2) with a set confidence threshold (0.8). Python scripts from Qiime (version 1.9.1) are utilized to compute OTU profiling tables and alpha/beta diversity analyses.

### Untargeted fecal metabolomic analysis

To an EP tube, 50 mg of sample was added with 1 mL of extract solution (containing methanol:acetonitrile: water = 2:2:1 along with isotopically labeled internal standard solution). After homogenizing samples for 4 min with 35 HZ, they were sonicated for 5 min in ice water. A cycle of homogenization and sonication was repeated three times and then the samples after 1 h incubation at a temperature of −40°C were subjected to centrifugation with 12,000 rpm for 15 min at 4°C. The resulting supernatants were transferred to a fresh glass vial for analysis. An equal aliquot of supernatant from all samples was used to make the quality control (QC) sample.

An LC-MS/MS analysis was performed using a UHPLC system (Vanquish, Thermo Fisher Scientific, Waltham, MA, United States) equipped with a UPLC BEH Amide column (2.1 mm × 100 mm, 1.7 μm) coupled to Q Exactive HFX mass spectrometer (Orbitrap MS, Thermo Fisher Scientific, Waltham, MA, United States). The mobile phase consisted of solutions A and B where A consists of 25 mmol/L ammonium acetate along with 25 mmol/L ammonium hydroxide in water (pH = 9.75) while B contains acetonitrile. The auto-sampler temperature has been set to 4°C, and the injection volume of 3 μL is used. The acquisition of MS/MS spectra is done utilizing the QE HFX mass spectrometer operated in IDA (information-dependent acquisition) mode under the control of acquisition software (Xcalibur, Thermo Fisher Scientific, Waltham, MA, United States). A full scan of the MS spectrum was also continuously monitored using acquisition software in IDA mode. In the ESI source conditions, sheath gas flow rate was set to 30 Arb, aux gas flow rate to 25 Arb, capillary temperature to 350°C, full MS resolution to 60,000, full MS/MS resolution to 7,500, collision energy to 10/30/60 in NCE mode and spray voltage set to 3.6 kV (positive) or −3.2 kV (negative).

The raw data were converted to the mzXML format using ProteoWizard and processed with an inhouse program, which was developed using R and based on XCMS, for peak detection, extraction, alignment, and integration (22). Then an inhouse MS2 database (Biotree DB) was applied in metabolite annotation. The cutoff for annotation was set at 0.3.

### Statistical analysis

The experimental data were expressed as mean ± SD. The differences between the two groups were analyzed using Student’s *t*-test. Similarly, the differences present in multiple groups were evaluated using one-way ANOVA. The Correlations found among variables were identified by Pearson’s product-moment correlation coefficient. Various tests were conducted using GraphPad Prism version 8.0 available for Windows (GraphPad Software, San Diego, CA, United States) and SPSS Statistics 26 (IBM Corporation, Armonk, NY). The *p* < 0.05 is considered an acceptable criterion of significance.

## Results

### Analysis of corn silk extract on the body weight, energy intake, and Glucose Tolerance Test

**FIGURE 1:**
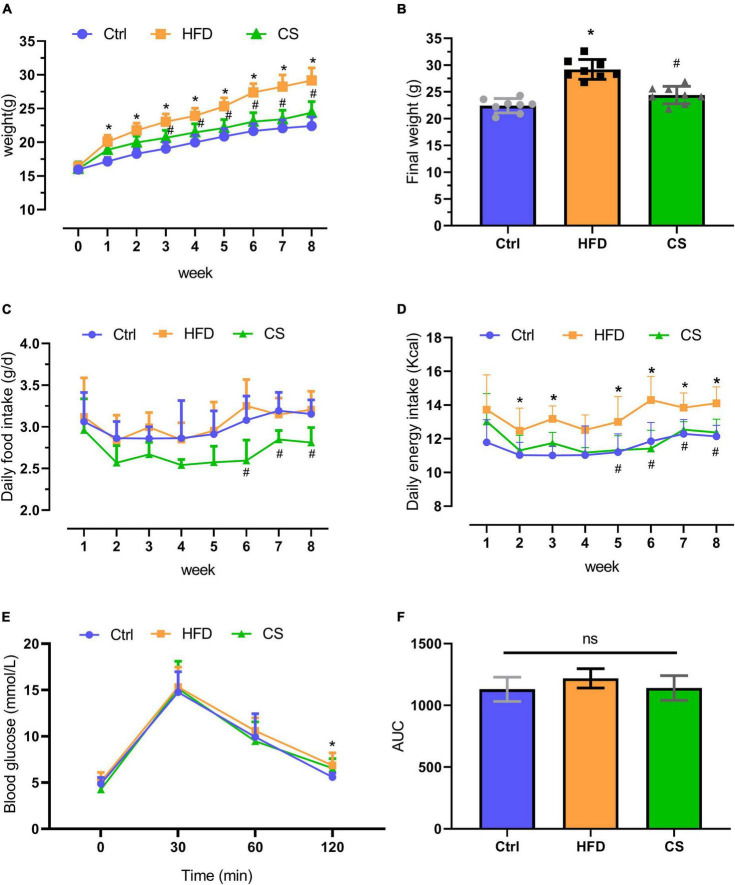
Effect of corn silk extract on body weight and energy intake. **(A)** Body weight; **(B)** final weight; **(C)** daily food intake; **(D)** daily energy intake; **(E)** Glucose Tolerance Test; **(F)** area under the curve of the Glucose Tolerance Test. Control diet (Ctrl) group, high- fat diet (HFD) group, corn silk (CS) group. The results are means ± SD (*n* = 8). *p < 0.05 vs. Ctrl, ^#^*p* < 0.05 vs. HFD. ns, not significant.

A dietary intervention of 8 weeks resulted in a remarkable increase in the body weight of mice in the HFD group (29.2 ± 1.9 g) in comparison to the mice in the control group (22.4 ± 1.3 g) (*p* < 0.05). However, supplementation with CS extract (24.4 ± 1.6 g) gradually reversed this HFD-induced increase in body weight (*p* < 0.05). The daily food intake of mice in the CS group was significantly lower at weeks 6, 7, and 8 compared to mice in the HFD group. The daily energy intake of mice in the CS group was significantly lower at weeks 5, 6, 7, and 8 compared to the HFD group (Figure 1). The Glucose Tolerance Test (GTT) assay indicated that the blood glucose of HFD mice was higher than that of control group at the 120 min time point (*p* < 0.05), and there was no significant difference in blood glucose and area under the curve (AUC) at each time point in the CS group compared with the HFD group (Figures 1E,F).

### Effect of corn silk extract on lipid levels

**FIGURE 2:**
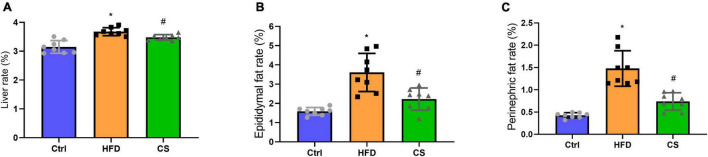
Effect of corn silk extract on liver, epididymal fat and perinephric fat rate (%). **(A)** liver rate; **(B)** epididymal fat rate; **(C)** perinephric fat rate. Control diet (Ctrl) group, high- fat diet (HFD) group, corn silk (CS) group. The results are means ± SD (*n* = 8). *p < 0.05 vs. Ctrl, ^#^*p* < 0.05 vs. HFD.

**FIGURE 3:**
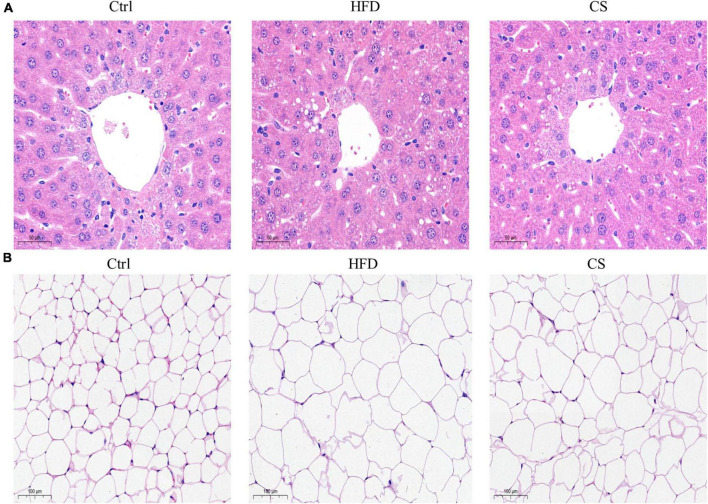
Effect of corn silk extract on histopathology of liver and adipose in mice. **(A)** Representative photomicrographs of hematoxylin and eosin-stained sections of the liver (400 × original magnification), **(B)** representative photomicrographs of hematoxylin and eosin-stained sections of the adipose (200 × original magnification). Control diet (Ctrl) group, high- fat diet (HFD) group, corn silk (CS) group.

According to Figure 2, weights of liver, epididymis fat, and perinephric fat tissues were significantly higher within the HFD group in comparison to the Control group, where the CS group prevented that increase (*p* < 0.05). Further, histology studies illustrated that the mice in the HFD group are having more severely degenerating hepatocytes, larger intracellular lipid droplets, and enlarged adipose tissue cells. In contrast, mice supplemented with CS extract showed reduced lipid load and tissue damage (Figure 3).

**FIGURE 4:**
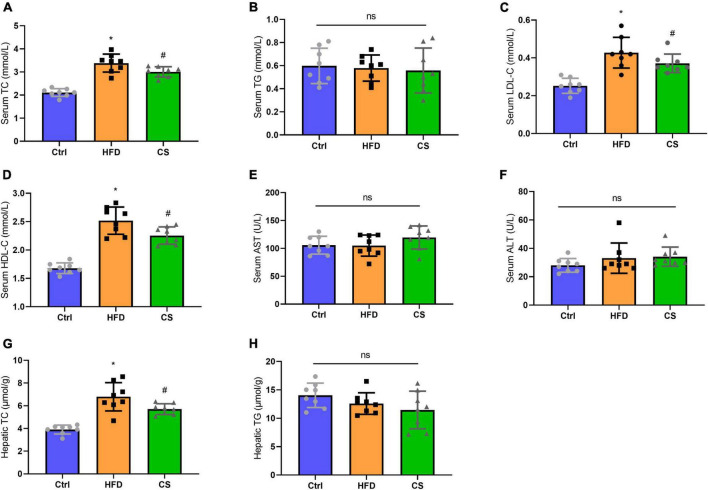
Effect of corn silk extract on lipid contents of the serum and liver. **(A)** Serum TC; **(B)** serum TG; **(C)** serum LDL- C; **(D)** serum HDL-C; **(E)** serum AST; **(F)** serum ALT; **(G)** hepatic TC; **(H)** hepatic TG. Control diet (Ctrl) group, high- fat diet (HFD) group, corn silk (CS) group. The results are means ± SD (*n* = 8). *p < 0.05 vs. Ctrl, ^#^*p* < 0.05 vs. HFD. ns, not significant.

Moreover, we also analyzed the lipid contents in the serum and liver. As compared to the control group, the serum and hepatic TC levels of the HFD group were both significantly higher at 3.38 ± 0.39 mmol/L and 6.79 ± 1.24 ± mol/g (*p* < 0.05), whereas the corresponding levels in CS group were considerably lower to 3.00 ± 0.22 mmol/L and 5.70 ± 0.474 μmol/g, respectively. Furthermore, the CS group also showed a decreasing trend in LDL-C levels. The HDL-C levels in the HFD group were considerably higher in comparison to the Control group (*p* < 0.05), however, CS extract supplementation reversed this trend. In terms of TG levels in the serum and liver, there were no differences between the groups. Additionally, we measured the ALT and AST activities of the mice and did not find any differences (Figure 4).

### Effect of corn silk extract on chronic inflammation

**FIGURE 5:**
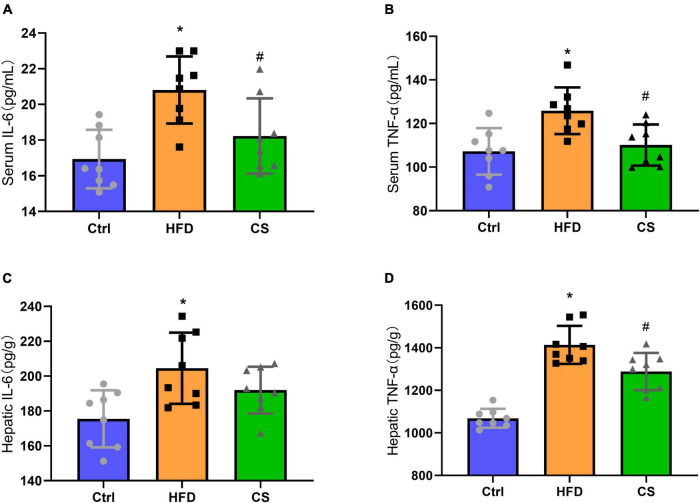
Effects of corn silk extract on chronic inflammation. **(A)** Serum IL-6; **(B)** serum TNF-α; **(C)** hepatic IL-6; **(D)** hepatic TNF-α. Control diet (Ctrl) group, high- fat diet (HFD) group, corn silk (CS) group. The results are means ± SD (*n* = 8). *p < 0.05 vs. Ctrl, ^#^*p* < 0.05 vs. HFD.

The anti-inflammatory abilities of CS extract were also determined in HFD mice and we found a significant increase in IL-6 and TNF-α levels of serum and liver in the HFD group in comparison to the control group indicating an occurrence of systemic chronic inflammation (*p* < 0.05). The IL-6 and TNF-α levels of serum and liver in the CS group displayed remarkably lowered levels compared with the HFD group but were not restored to the levels observed in control group (Figures 5A–D, *p* < 0.05).

### Effect of corn silk extract on gut microbiota diversity and composition

**FIGURE 6:**
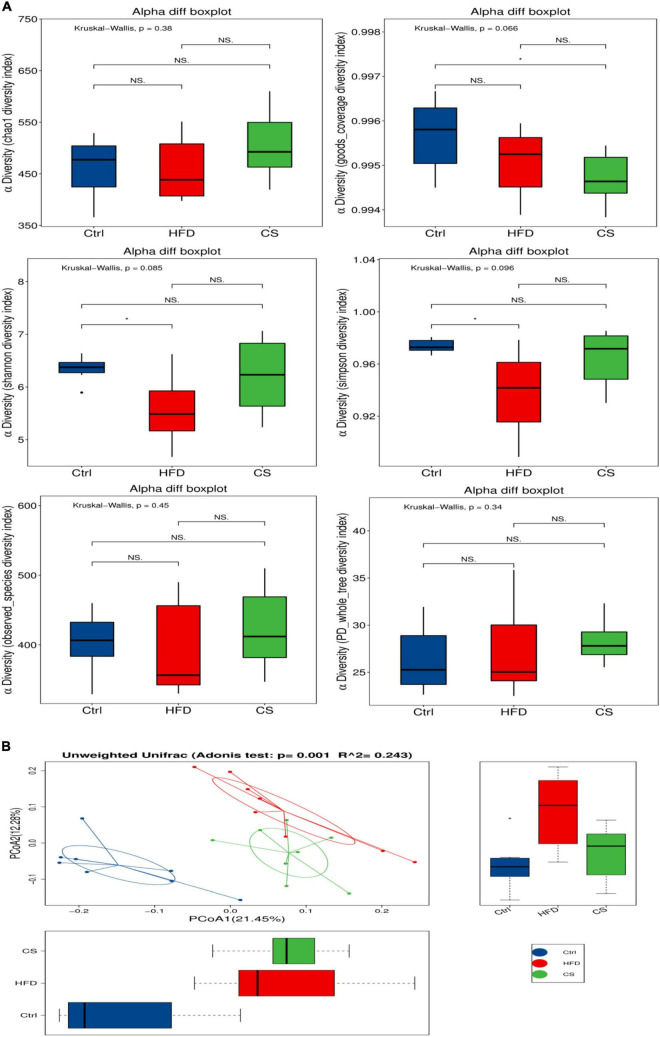
Effects of corn silk extract on the α- diversity and β- diversity index of gut microbiota in mice. **(A)** α-Diversity; **(B)** unweighted UniFrac PCoA analysis. Control diet (Ctrl) group, high- fat diet (HFD) group, corn silk (CS) group. The results are means ± SD (*n* = 8). *p < 0.05 vs. other group. ns, not significant.

For each group (*n* = 8), freshly collected feces samples were used to determine the microbiota profile. A total of 856,559 clean reads were picked up after quality filtering where they had an average length of 416 bp. Moreover, clustering of these clean reads into 1,129 OTUs was done using a similarity level of 97%. Figure 6 illustrates the α-diversity and β-diversity indexes of gut microbiota in mice. A lower level of Simpson, Shannon diversity indexes have been found among the HFD group in comparison to the control group, however, the CS group had no obvious effects. Both the Simpson and Shannon diversity indexes are used to assess the diversity of microbial communities, with higher indexes resulting in higher diversity. To further demonstrate the variation in species diversity between samples, Principal Coordinates Analysis (PCoA) was used to demonstrate the magnitude of variation between samples. PCoA analysis of species diversity between samples, where two samples are close together, indicates that the species composition of the two samples is similar. Adonis analysis, also known as PERMANOVA (Permutational multivariate analysis of variance) analysis, can decompose the total variance using a semimetric (e.g., Unifrac) or metric distance matrix (e.g., Euclidean) to analyze, through a linear model *R*-values indicate the dilution of the variance of the sample by group or environmental factor, i.e., the ratio of the variance of the group or environmental factor indicator to the total variance. The *p*-values indicate the confidence level of this analysis, adnois analysis is often combined with PCoA. A UniFrac PCoA analysis revealed further that the gut microbiota composition of mice in HFD group and CS group were both distinct from the control group (Figure 6, *p* < 0.05).

**FIGURE 7:**
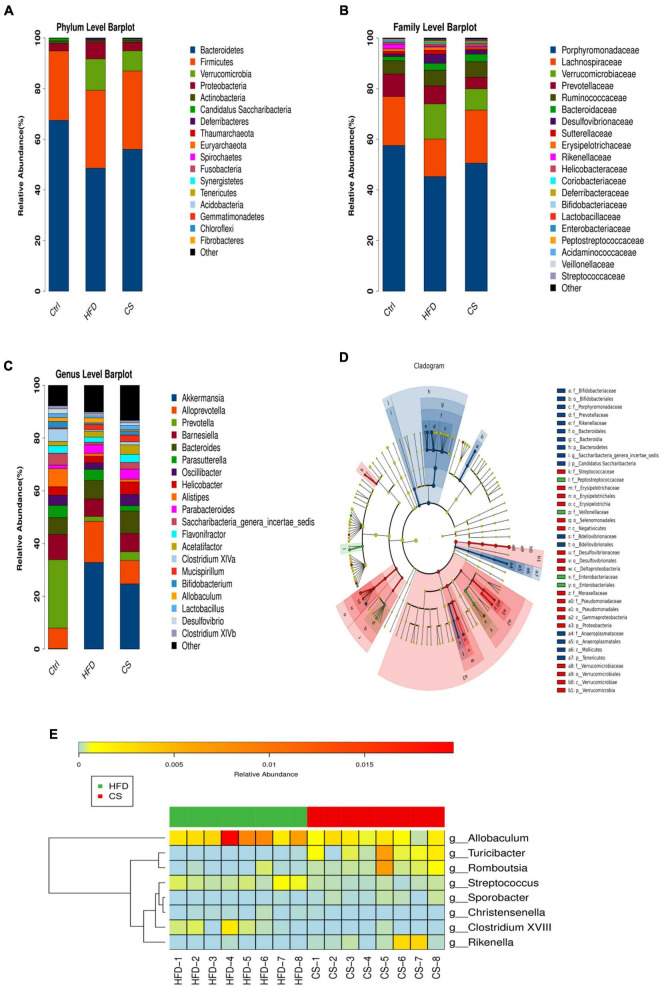
Comparison of community composition. **(A)** Microbial community bar plot at the phylum level; **(B)** microbial community bar plot at the family level; **(C)** microbial community bar plot at the genus level; **(D)** LDA effect size; **(E)** heatmap of significant differences microbiota at the genus level. Control diet (Ctrl) group, high- fat diet (HFD) group, corn silk (CS) group.

In mice, *Bacteroidetes, Firmicutes, Verrucomicrobia*, and *Proteobacteria* were found to be dominant at the phylum level (Figure 7A). When compared to the control group, the consumption of HFD prompted the abundance of *Firmicutes* (30.76% vs. 27.43%), *Verrucomicrobia* (12.40% vs. 0.04%), and *Proteobacteria* (6.67% vs. 2.89%), but inhibited the abundance of *Bacteroidetes* (48.57% vs. 67.45%). On the other hand, the CS group increases the abundance of *Bacteroidetes* while reducing *Verrucomicrobia* and *Proteobacteria*. At the family level, the mice belonging to the HFD group had a higher abundance of *Verrucomicrobiaceae* (13.91% vs. 0.04%), *Bacteroidaceae* (2.71% vs. 1.64%), and *Desulfovibrionaceae* (3.54% vs. 0.92%), and a lower abundance of *Porphyromonadaceae* (45.25% vs. 57.51%) and *Lachnospiraceae* (14.83% vs. 19.41%) compared to the control group (Figure 7B). The intake of CS extract, however, reversed the gut microbiota imbalance as a result of HFD. At a genus level, the changes in the fecal microbiota community were shown in Figure 7C. The results of the LEfSe analysis using 2.0 as a threshold on the LDA score were shown in Figure 7D. Different colors correspond to different groupings, while nodes of different colors represent microbial groups that play an important role in that grouping. There are yellow nodes representing microorganisms that do not have much of an impact on other groups. Furthermore, our studies at the genus level showed significant differences in microbiota including *Allobaculum*, *Turicibacter*, *Romboutsia*, *Streptococcus*, *Sporobacter*, *Christensenella*, *Clostridium XVIII*, and *Rikenella* in HFD and CS group (*p* < 0.05, Figure 7E).

**FIGURE 8:**
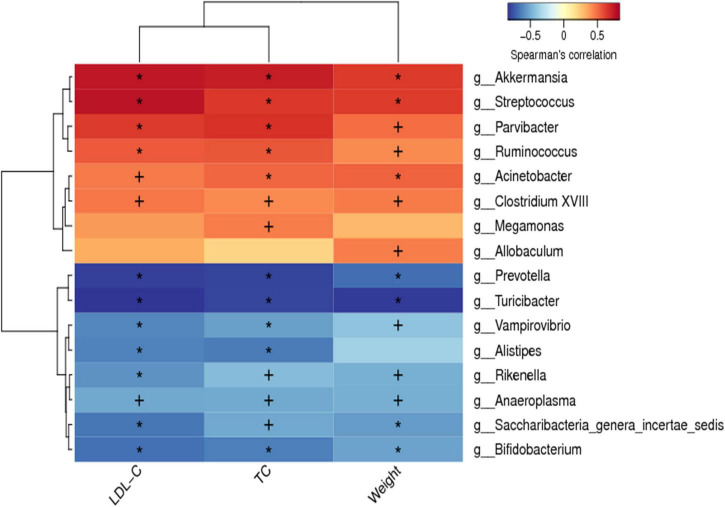
Spearman correlations between hypercholesterolemia symptoms and gut microbiota. Positive correlations indicated by red cubes and negative correlations indicated by blue cubes. At the same time, the correlation significant test was carried out, when *p* < 0.05, + was used to mark the significance, when *p* < 0.01, * was used to mark the significance.

Spearman correlation analysis was conducted between the dominant bacteria at the genus level of all groups and hypercholesterolemia symptoms to identify specific bacteria that attenuate hypercholesterolemia symptoms. The horizontal coordinates represent the environmental factors and the vertical coordinates represent the species, the shades of color visually show the correlation between the species and the environmental factors, where red indicates positive correlation and blue indicates negative correlation. The correlation is also tested for significance, with + marked as significant when *p* < 0.05 and * marked as significant when *p* < 0.01. A positive correlation between the genus and hypercholesterolemia symptoms indicates that an increase in the abundance of the genus may promote the development of hypercholesterolemia, while a negative correlation indicates that an increase in the abundance of the genus may favor the suppression of hypercholesterolemia. As shown in Figure 8, the abundance of *Akkermansia, Streptococcus, Parvibacter, Ruminococcus, Acinetobacter*, and *Clostridium XVIII* were significantly positively correlated to the body weight, serum TC and LDL-C (*p* < 0.05). Besides, the abundance of *Prevotella, Turicibacter, Vampirovibrio, Rikenella, Anaeroplasma, Saccharibacteria-genera-incertaesedis*, and *Bifidobacterium* were found to have a significant negative correlation with the weight of the body, and levels of serum TC and LDL-C (*p* < 0.05). Although the plethora of *Megamonas* and *Allobaculum* were linked to serum LDL-C levels, however, they were not statistically significant (*p* > 0.05).

### Effect of corn silk extract on untargeted fecal metabolomic analysis

We collected fresh feces samples from each group (*n* = 8) for analysis of the effects of CS extract on HFD mice using UHPLC-QE HFX-MS. LC-MS data were analyzed using PCA and OPLS-DA to determine metabolic changes. Based on the metabolic profiles in Supplementary Figure 1, the PCA and OPLS-DA score plots distinguished between the control and HFD groups for positive (POS) as well as negative (NEG) modes. Control and HFD groups achieved an R^2^Y and Q^2^ of respectively 0.999 and 0.992 in the POS ion mode and 0.998 and 0.985 in the NEG ion mode (Supplementary Figure 1C). Both groups (CS and HFD) underwent thorough separation in POS as well as NEG ion modes (Supplementary Figure 2). R^2^Y and Q^2^ were 0.96 and 0.709, respectively, for POS mode as well as 0.895 and 0.597, respectively, for the NEG ion mode. In all cases, R^2^Y values were > 0.5, indicating a reliable and stable model. The outcomes from PCA along with OPLS-DA supported that establishment of the HFD mice model is successful as well as the ability of CS extract supplementation in regulating metabolic profiles of the mice.

A volcano plot was used to compare potential biomarkers between the two groups. Then, we conducted a Student’s *t*-test (Supplementary Figure 3). On the volcanic map, each point denoted a metabolite where its size portrays the OPLA-DA model’s VIP value (variable importance in projection). Metabolites that are considerably up-regulated, down-regulated, and non-significant are shown as colors (red, blue, and gray). Using a commercial database being self-established as a base, screening of differential metabolites was achieved with thresholds of VIP > 1 and *p* < 0.05. The HMDB and KEGG databases data matched the model of MS/MS fragmentation that furnish information on metabolites structure. A total of 583 metabolites were identified through heatmaps generated with analysis of hierarchical clustering between control and HFD groups (Supplementary Figure 4), including 478 and 105 metabolites in the POS and NEG ion modes, respectively. The CS and HFD groups identified 201 metabolites, of which 171 metabolites were identified in the POS ion mode and 30 in the NEG ion mode.

**FIGURE 9:**
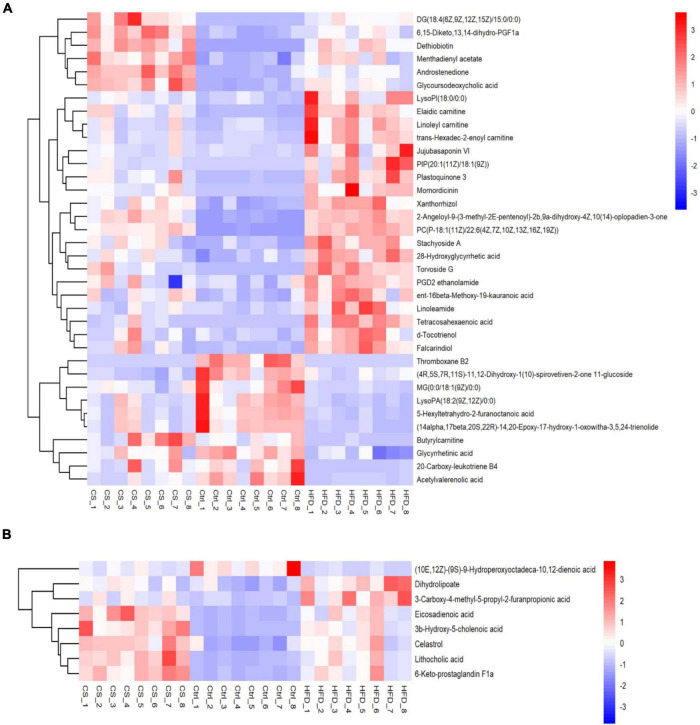
Heatmap of lipids and lipid-like molecules shared by all groups (**A:** POS ion, **B:** NEG ion). Control diet (Ctrl) group, high- fat diet (HFD) group, corn silk (CS) group.

In total, 44 substances made up of lipids and lipid-like molecules shared by all groups were screened, as shown in Figure 9. For the HFD group, the number of metabolites detected was larger in comparison to control group, especially Xanthorrhizol, Butyrylcarnitine, 2-Angeloyl- 9-(3-methyl-2E-pentenoyl)-2b,9a-dihydroxy-4Z,10(14)-oplopadien-3-one, d-Tocotrienol, Plastoquinone 3, Linoleamide, Jujubasaponin VI, ent-16beta- Meth-oxy-19-kauranoic acid, 28-Hydroxyglycyrrhetic acid, PGD2 ethanolamide, Tetraco-sahexaenoic acid, Elaidic carnitine, PIP[20:1(11Z)/18:1(9Z)], Stachyoside A, Falca-rindiol, PC[P-18:1(11Z)/22:6(4Z,7Z,10Z,13Z,16Z,19Z)], Torvoside G, LysoPI(18:0/0:0), Linoleyl carnitine, trans-Hexadec-2-enoyl carnitine, and Momordicinin. In contrast, those with lower levels in the HFD group than in Ctrl group included 20-Carboxy-leukotriene B4, Acetylvalerenolic acid, Glycyrrhetinic acid, 5-Hexyltetrahydro-2-furanoctanoic acid, LysoPA[18:2(9Z,12Z)/0:0], MG[0:0/18:1(9Z)/0:0] and (14alpha,17beta,20S,22R)-14,20-Epoxy-17-hydroxy-1-oxowitha-3,5,24-trienolide. Whereas supplementation of CS extract reversed the changes caused by HFD.

### KEGG metabolic pathway analysis

**FIGURE 10:**
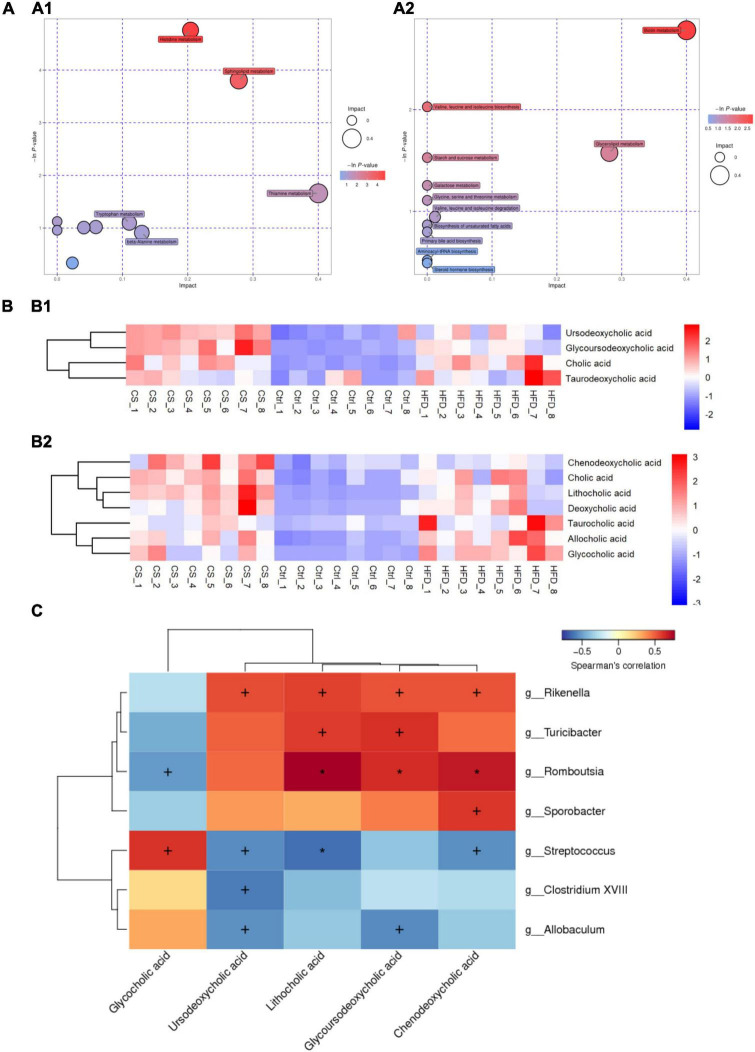
CS supplementation modulates fecal metabolite pathways and profiles in HFD-fed mice. **(A)** Bubble plot map of metabolic pathways (A1: POS ion, A2: NEG ion); **(B)** heatmap of bile acids (B1: POS ion, B2: NEG ion); **(C)** heatmap of Spearman’s correlation between microbiota and bile acids, the correlation significant test was carried out, when *p* < 0.05, + was used to mark the significance, when *p* < 0.01, * was used to mark the significance. Control diet (Ctrl) group, high- fat diet (HFD) group, corn silk (CS) group.

Potential metabolic pathways were analyzed using the differential metabolites screened above. The identified metabolites were classified by Human Metabolome Database (HMDB) into 29 subclasses, such as amino acid, bile acid, aromatic compounds, flavonoids, neurotransmitters, alkaloids, carbohydrates, vitamins, organic acid, fatty acids, fatty acyls, plant hormone, etc. KEGG data were used for pathway enrichment analysis and bubble plot map depicted as shown in Figure 10A. Here, the bubble size was positively correlated with the influence factor size of this pathway in topological analysis. An enrichment analysis *p*-value is represented by the color of the bubble: darker color reflects a smaller *p*-value with a greater degree of enrichment. Ten candidate metabolic pathways in the POS ion mode being regulated after CS extract treatment were as follows: Histidine metabolism; Sphingolipid metabolism; Thiamine metabolism; Nicotinate and nicotinamide metabolism; Tryptophan metabolism; Steroid hormone biosynthesis; Pantothenate and CoA biosynthesis; Pentose and glucuronate interconversions; beta-Alanine metabolism and Pyrimidine metabolism. Meanwhile, 11 potential metabolic pathways in the NEG ion mode were found: Biotin metabolism; Valine, leucine, and isoleucine biosynthesis; Glycerolipid metabolism; Starch and sucrose metabolism; Galactose metabolism; Glycine, serine, and threonine metabolism; Valine, leucine, and isoleucine degradation; Biosynthesis of unsaturated fatty acids; Primary bile acid biosynthesis; Aminoacyl-tRNA biosynthesis and Steroid hormone biosynthesis.

Furthermore, the abundance of primary bile acids including cholic acid (CA), chenodeoxycholic acid (CDCA), glycocholic acid (GCA), and taurocholic acid (TCA), as well as secondary bile acids including deoxycholic acid (DCA), lithocholic acid (LCA), taurodeoxycholic acid (TDCA), Ursodeoxycholic acid (UDCA), glycoursodeoxycholic acid (GUDCA), and allocholic acid (ACA) in the three groups were shown in Figure 10B. HFD increased the abundance of bile acids in the feces of mice, however, the levels of CDCA, LCA, UDCA, and GUDCA were significantly higher after the CS extract intervention compared to the HFD group. Thus, CS extract supplementation affected bile acid metabolism. Using Spearman’s correlation analysis, we also calculated the correlations between the substantial differences in gut microbiota at the genus level and the amounts of bile acids. As shown in Figure 10C, the abundance of *Rikenella*, *Turicibacter*, *Romboutsia*, and *Sporobacter* were positively correlated to the amounts of CDCA, GUDCA, LCA, and UDCA. In contrast, the abundance of *Streptococcus*, *Clostridium XVIII*, and *Allobaculum* had a negative correlation with the amounts of CDCA, GUDCA, LCA, and UDCA. Besides, the amounts of GCA was positively correlated to the abundance of *Streptococcus*, *Clostridium XVIII*, and *Allobaculum*, and negatively correlated with the abundance of *Rikenella*, *Turicibacter*, *Romboutsia*, and *Sporobacter*.

## Discussion

In the present study, corn silk extract was investigated for its effects on body weight, lipid levels, intestinal microbiota, and fecal metabolomics in HFD-fed mice. There is a clear link between obesity and hypercholesterolemia. In HFD-fed mice, the CS extract significantly decreased body weight, adipose tissue weight, and liver weight. As a vital organ to maintain cholesterol homeostasis, the liver is responsible for not only cholesterol *de novo* synthesis but also biliary cholesterol elimination.

To rule out the possibility that the reduced body weight and liver size are a consequence of liver damage induced by CS extract treatment, we measured serum ALT and AST activity levels of the HFD mice. As a result, CS extract did not impair liver function. Furthermore, the serum and hepatic levels of TC in mice were also significantly reduced by CS extract. Previous reports showed that the CS reduced adipose tissue weight and improved cholesterol metabolism in mice fed HFD (18, 23), and the benefit is likely due to the inhibition of genes involved in adipocyte differentiation, fat accumulation, and fat synthesis (24). Mice of the HFD group also exhibited macrovesicular steatosis around the central vein of hepatocytes as well as enlarged adipocytes in the adipose tissue of the epididymis. However, the CS extract ameliorated these morphological changes. Based on these results, the CS extract exerted a protective effect on HFD-fed mice by lowering weight and lipid levels.

The adipose tissue does not only contain adipose cells but also macrophages, which are closely associated with inflammation. It has been found that obesity can increase both local and systemic inflammation, whereas obesity is a chronic low inflammation caused by various inflammatory factors (25). In an attempt to determine whether the CS extract could alleviate inflammation, we studied the change in inflammation. Macrophages are major immune effector cells that release inflammatory cytokines. These cytokines include tumor necrosis factor α (TNF-α) and interleukin 6 (IL-6) (26). The result showed that supplementing with CS extract reduced serum and liver levels of TNF-α and IL-6. Thus, CS extract may be responsible for alleviating liver injury by reducing HFD-induced liver inflammation.

By analyzing high throughput sequencing data, we determined the effects of CS extract on fecal microbiota composition and structure. Another study highlighted the crucial role of gut microbiota in metabolic diseases, such as obesity and diabetes (27). In the present study, we found that HFD drastically changes the gut microbiota composition of C57BL/6J mice. The administration of HFD to mice reproducibly induces obesity, metabolic syndrome, and hepatic steatosis (28). The metabolism of the host is highly influenced by the microbiota in the intestine, the composition and function of the microbiota are dynamic and affected by dietary characteristics such as the amount and composition of lipids. Dietary lipids may therefore alter host physiology by interacting with gut microbiota (29). Gut microbiota is affected by lipids both as substrates for bacterial metabolic processes and inhibits bacterial growth. Mice and humans have been shown to have altered lipid metabolism and lipid levels in their blood and tissues due to their gut microbes (30). The gut microbiota profile is affected by conditions such as dyslipidemia, hyperlipidemia, non-alcoholic liver disease, and atherosclerosis (31, 32).

HFD increased the abundance of *Firmicutes*, *Verrucomicrobia*, and *Proteobacteria*, but inhibited the counts of *Bacteroidetes* at the phylum level. Meanwhile, the CS group increases the abundance of *Bacteroidetes* and decreases the abundance of *Verrucomicrobia* and *Proteobacteria*. Previous studies suggest the change in the load of *Firmicutes* and *Bacteroidetes* may be a marker for metabolic disorder in HFD animal models and can be exploited for research benefits (33, 34). However, some studies suggest that linking Firmicutes/Bacteroidetes ratio with health status is currently difficult (35, 36).

The LEfSe analysis noted significant differences in microbiota between HFD and CS groups at the genus level, including *Allobaculum*, *Turicibacter*, *Romboutsia*, *Streptococcus*, *Sporobacter*, *Christensenella*, *Clostridium XVIII*, and *Rikenella*. Subsequently, our findings found an association between significant differences in microbiota and symptoms of hypercholesterolemia (Figure 8). *Allobaculum* cells exhibit a shape like a rod and are Gram-positive in staining with a paired or chain arrangement (37). Researchers found that *Allobaculum* produced butyric acid and is linked positively to Angiogenin-like protein 4 (ANGPTL4) expression, which is regarded to be a circulating mediator between the microbiota of gut and fat storage (38–40). Butyric acid transactivated PPARγ and regulated ANGPTL4, the target gene of PPARγ, in colon cells (41). FIAF (fasting induction factor), another term for ANGPTL4, is reported to regulate the metabolism of lipids while playing a crucial role in the deposition of lipids via inhibition of lipoprotein lipase (LPL) (42). Studies revealed that the abundance of *Allobaculum* might be linked reversely to inflammation, insulin resistance, and obesity with the intervention of berberine and metformin in mice (43). In the present study, CS extract supplementation reduced the relative abundance of *Allobaculum* in the GM of HFD mice, which was beneficial for the inhibition of inflammation and the regulation of lipid metabolism. Many studies have reported *Turicibacter* as an anti-inflammatory taxon due to its inverse relationship with NF-B1 protein (44–46). Further, the results of other scientists indicate that the abundance of *Turicibacter* is markedly reduced in mice fed HFD, and there is a negative correlation between the abundance of *Turicibacter* and body weight (47–50), this is consistent with the results observed in our experiments, and after intervention with CS extract, the abundance of *Turicibacter* was significantly higher, which contributed to the anti-inflammatory effect. *Turicibacter* inhabits the small intestine and deconjugates 96∼100% of glycochenodeoxycholic acid and taurocholic acid within 24 h after meals when BAs are released. In addition, BA levels may affect the abundance of *Turicibacter* (51). In the human microbiome, *Streptococcus* is ubiquitous and belongs to the phylum *Firmicutes* (52). *Streptococci* are often considered to be obligate or opportunistic pathogens that cause a wide variety of diseases in various organs, such as pneumonia, endocarditis, and meningitis (53, 54). *Streptococcus* abundance in feces may be reduced due to the anti-inflammatory effect of CS extract. The gut microbiota of animals fed CS extracts is enriched with *Sporobacter* compared to animals fed a HFD. In addition, the observed anti-hyperlipidemic effect of *Rhizoma Coptidis* alkaloids could also be attributed to the promotion of the abundance of *Sporobacter termitidis*, *Alcaligenes faecalis*, and *Akkermansia muciniphila* in obese mice (55).

In the present study, reduced abundance of *Christensenella* was found in the feces of CS extract-fed mice and it was suggested that the reduced abundance of *Christensenella* had a beneficial effect in alleviating hypercholesterolemia. However, family *Christensenellaceae* is known to be negatively associated with obesity, hypertriglyceridemia and body mass index (BMI) and to have a potentially beneficial effect on gut microbes, and in humans with high levels of *Christensenellaceae*, the levels of lipid biosynthesis and energy metabolism pathways are reduced (56, 57). It is a fact that the diversity of microbes exists even within the same family, so uniqueness can only be guaranteed at the strain level. Meanwhile, other studies have reported that an unknown genus belonging to the *Christensenellaceae* was found to be negatively associated with weight loss (58), and an OTU was annotated as a member of the family *Christensenellaceae*, whose representative sequence had the highest identity with *Christensenella*, but with a positive correlation of obesity (59). Studies have shown that *Clostridium XVIII* produces exotoxins and promotes inflammation (60, 61), however, some studies have observed their potential ability to induce homeostatic T-reg responses (62). In response to cancer, *Clostridium XVIII* may additionally activate Tregs and increase the risk of tumors (63). It has been demonstrated that prolonged feeding of HFD will increase the abundance of *Clostridium XVIII* and the degree of inflammation, while *Clostridium XVIII* showed significant correlations with serum and hepatic lipid profiles (64). HFD mice fed by CS extract had a significant decrease in the abundance of *Clostridium XVIII* in GM. *Rikenella* is a genus of the family *Rikenellaceae*, the GM of overweight/obese patients exhibited a remarkable reduction in the relative abundance of *Rikenella* in comparison to normal-weight controls (65). Consistent with the results of this study, the HFD-induced reduction in *Rikenella* abundance was alleviated by supplementing with CS extract. However, some studies have shown that HFD increases the abundance of *Rikenella* in male C57BL/6J mice (66, 67), but there are also opposite results (68). This suggests that the abundance of *Rikenella* may be altered by changes in the intestinal environment and that it may not have a potential role in itself. To a certain extent, our current research indicates that GM changes induced by HFD and reversed by CS extract may explain the ameliorating effects of CS extract on hypercholesterolemia.

Both the host and the gut microbiota produce fecal metabolites that play an important role in preserving health. To find out the differential metabolites involved in the beneficial effect of CS extract, we analyzed the changes in metabolite profile and metabolic pathway by using feces samples. The histidine metabolism pathway, which contains three differential metabolites urocanic acid, methylimidazole acetaldehyde, and methylimidazoleacetic acid, showed the most significant enrichment (Figure 10A). Histidine is an essential amino acid for mammals as it cannot be synthesized by the body *de novo* and must be obtained from the diet (69). Studies have shown that histidine reduces body fat, decreases appetite, increases insulin sensitivity, reduces oxidative stress, and lowers levels of systemic inflammation markers in plasma (70). Based on metabolic pathway enrichment analysis, sphingolipid metabolism and biotin metabolism were also significantly altered. Sphingolipid metabolism is significantly affected by HFD (71). Sphingolipids have biologically significant roles in nearly all aspects of cell biology, including cell death, the cell cycle, immune responses, inflammation, angiogenesis, nutrient uptake, metabolism, stress responses, and autophagy (72). The balance between biotin metabolism and recycling is essential to normal bacterial growth and function in the microbiota, and altered biotin metabolism is associated with obesity-related metabolism and systemic inflammation (73). On the other hand, we found that CS extract had a significant effect on bile acid biosynthesis and increased bile acid levels in feces, including CDCA, LCA, UDCA, and GUDCA. Alterations in bile acid metabolism directly affect hepatic metabolic homeostasis and contribute to the development of metabolic diseases such as non-alcoholic fatty liver disease (NAFLD), obesity, and inflammatory bowel disease (IBD) (74). Bile acids are known to produce from cholesterol in the liver, and considered the primary lipid component of bile, and have the capacity to bind to taurine or glycine for enhancing its solubility. Subsequently, there is a secretion of BAs in bile resulting in its concentration and storage in the gallbladder. The intake of food causes a release of bile into the duodenum from the gallbladder, thereby making BAs accessible to lipids and fat-soluble vitamins assisting in their digestion and absorption, respectively (75). Bile acids regulate the absorption of fats and steroids in the gut and act as signaling molecules activated by farnesoid X receptor (FXR) and G-protein-coupled bile acid receptor-1 (GPBAR-1) in the liver, intestine, muscle, and brown adipose tissue (76, 77). In the liver, cholesterol is catabolized by the conversion of cholesterol to bile acids. In the meantime, the bile acids’ re-entry to the liver via enterohepatic circulation will affect the production of cholesterol in the liver. The ileum is the site for the passive absorption of bile acids, but these acids are mostly reabsorbed into the portal blood circulation with the active transport system, and then to the liver, where small amounts of bile acids are excreted in the feces are replaced by *de novo* synthesis in the liver. Consequently, with increased bile acid loss in the feces, cholesterol will be converted to bile acids in the liver, lowering cholesterol levels.

## Conclusion

In conclusion, the CS extract intervention improved the hypercholesterolemia caused by HFD and the potential mechanisms may include: (1) improved inflammation of the liver; (2) regulating the abundance of certain functional gut microbiota, including *Allobaculum*, *Turicibacter*, *Sporobacter*, and *Clostridium XVIII*; (3) exert an impact on the primary bile acid biosynthesis. A follow-up study using germ-free mice and fecal microbiota transplants will provide us with enhanced insights into the mechanism through which CS affects the microbiota in the gut, thus evaluating the role of metabolite-microbe interactions on the metabolic health of the host.

## Data availability statement

The 16S rRNA gene sequences were provided and available at NCBI Sequence Read Archive (SRP) repository with Accession Code: PRJNA817057; untargeted metabolomic data have been deposited to the EMBL- EBI MetaboLights database with the identifier MTBLS4543, the complete data set can be accessed at www.ebi.ac.uk/metabolights/MTBLS4543.

## Ethics statement

The animal study was reviewed and approved by the Animal Ethical Care Committee of Qiqihar Medical University.

## Author contributions

JL and HG designed the study. LD and SR conducted the animal trial, sample collection, and physical analysis. LD and HG performed the bioinformatics analysis of 16S rRNA sequencing and untargeted metabolomics data. LD and JL wrote the manuscript. YL, HG, CZ, YS, and WY contributed to the discussion of the work and assisted in drafting the manuscript. All authors read and approved the final manuscript.

## Footnotes

1
http://drive5.com/uparse/

2
http://rdp.cme.msu.edu/
