# Supplementary material for: Modulation of gut microbiota and fecal metabolites by corn silk among high-fat diet-induced hypercholesterolemia mice
Source: Front Nutr. 2022 Aug 1;9:935612. doi: 10.3389/fnut.2022.935612 (PMC9376456; doi:10.3389/fnut.2022.935612)
Supplement: Supplementary file 1 [file Table_1.DOCX]

Supplementary Material

# Supplementary Figures

**Supplementary Figure 1**. PCA and OPLS-DA analysis of feces for group Ctrl vs HFD of mice. (A) Score scatter plot of PCA model (A1: POS ion, A2: NEG ion); (B) Score scatter plot of OPLS-DA model (B1: POS ion, B2: NEG ion); (C) Permutation test of OPLS-DA model (C1: POS ion, C2: NEG ion). Control diet (Ctrl) group, high- fat diet (HFD) group, corn silk (CS) group.

**Supplementary Figure 2**. PCA and OPLS-DA analysis of feces for group CS vs HFD of mice. (A) Score scatter plot of PCA model (A1: POS ion, A2: NEG ion); (B) Score scatter plot of OPLS-DA model (B1: POS ion, B2: NEG ion); (C) Permutation test of OPLS-DA model (C1: POS ion, C2: NEG ion). Control diet (Ctrl) group, high- fat diet (HFD) group, corn silk (CS) group.

**Supplementary Figure 3**. Volcano plot of comparison groups: Ctrl vs HFD (A1: POS ion, A2: NEG ion); CS vs HFD (B1: POS ion, B2: NEG ion). Control diet (Ctrl) group, high- fat diet (HFD) group, corn silk (CS) group.

**Supplementary Figure 4**. Effects of corn silk extract on fecal metabolites. (A) Heatmap of hierarchical clustering analysis for group Ctrl vs HFD (A1: POS ion, A2: NEG ion); (B) Heatmap of hierarchical clustering analysis for group CS vs HFD (B1: POS ion, B2: NEG ion). Control diet (Ctrl) group, high- fat diet (HFD) group, corn silk (CS) group.
