# Supplementary figures and images for: Modulation of gut microbiota and fecal metabolites by corn silk among high-fat diet-induced hypercholesterolemia mice
Source: Front Nutr. 2022 Aug 1;9:935612. doi: 10.3389/fnut.2022.935612 (PMC9376456; doi:10.3389/fnut.2022.935612)

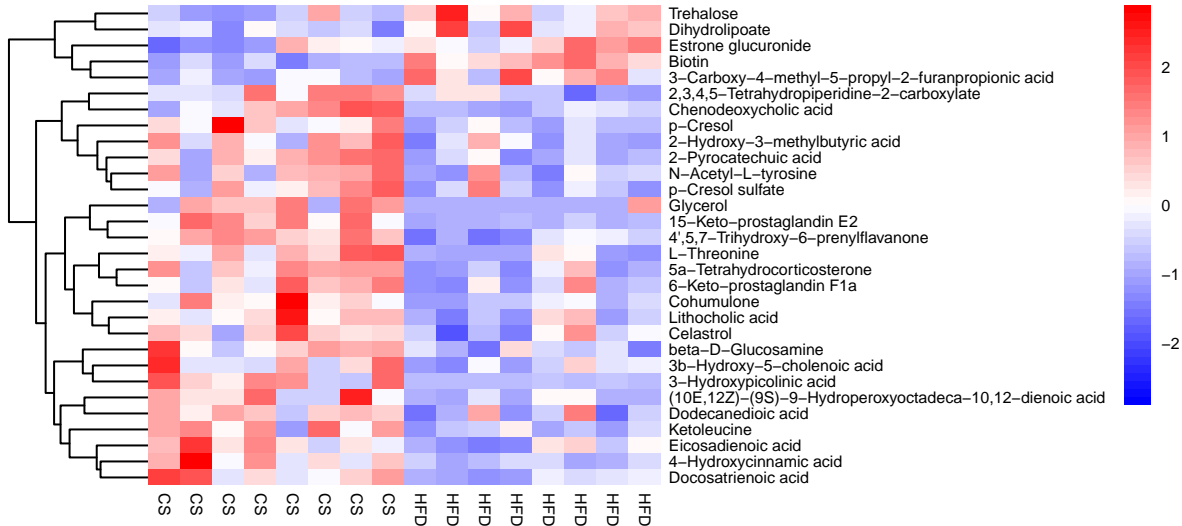

Supplement: Supplementary file 2 [file Data_Sheet_1.PDF]

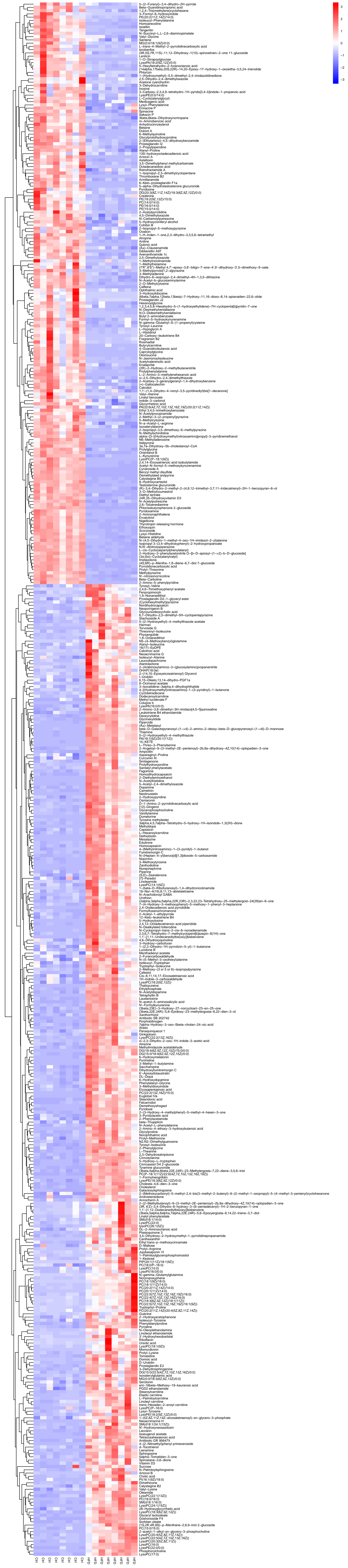

Supplement: Supplementary file 3 [file Data_Sheet_2.PDF]

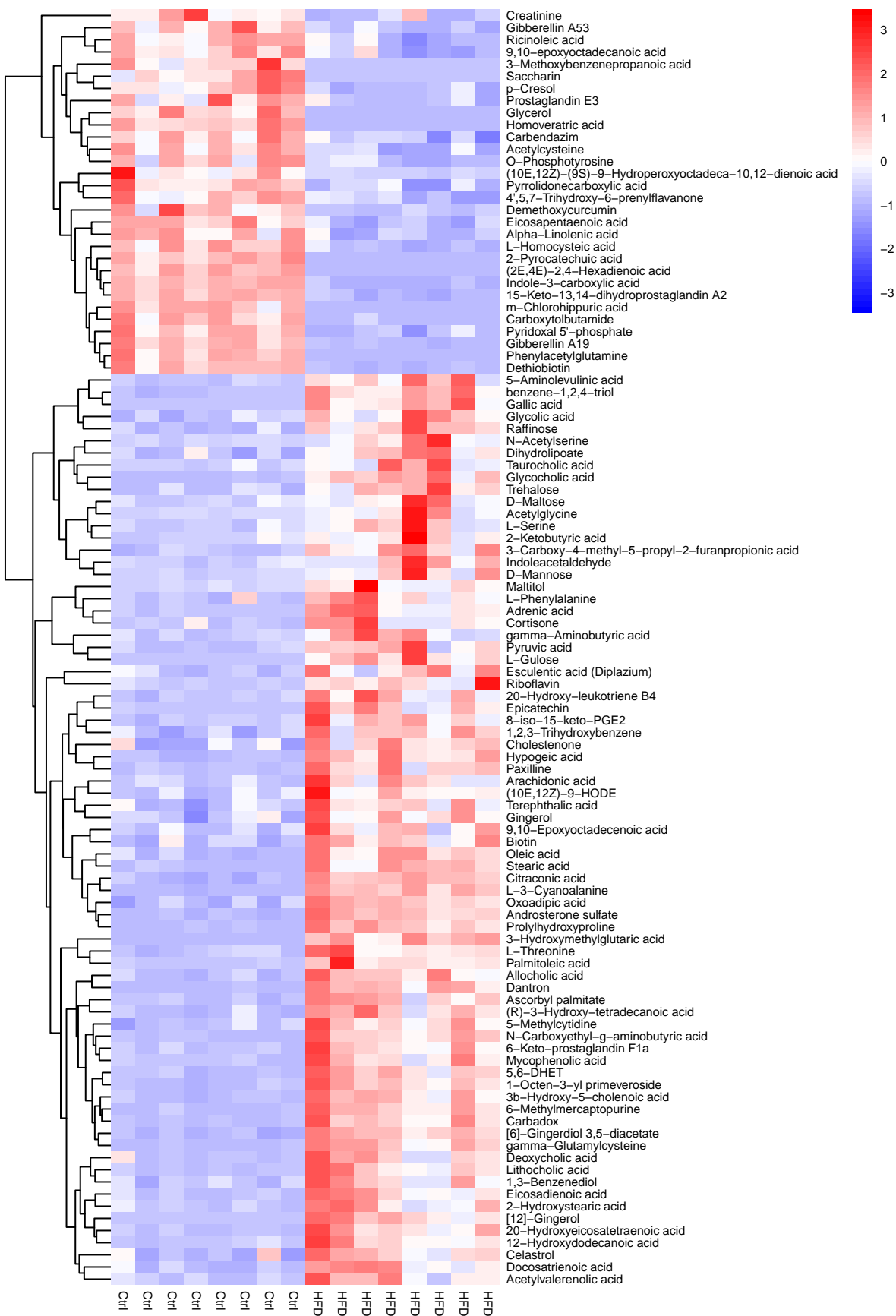

Supplement: Supplementary file 4 [file Data_Sheet_3.PDF]

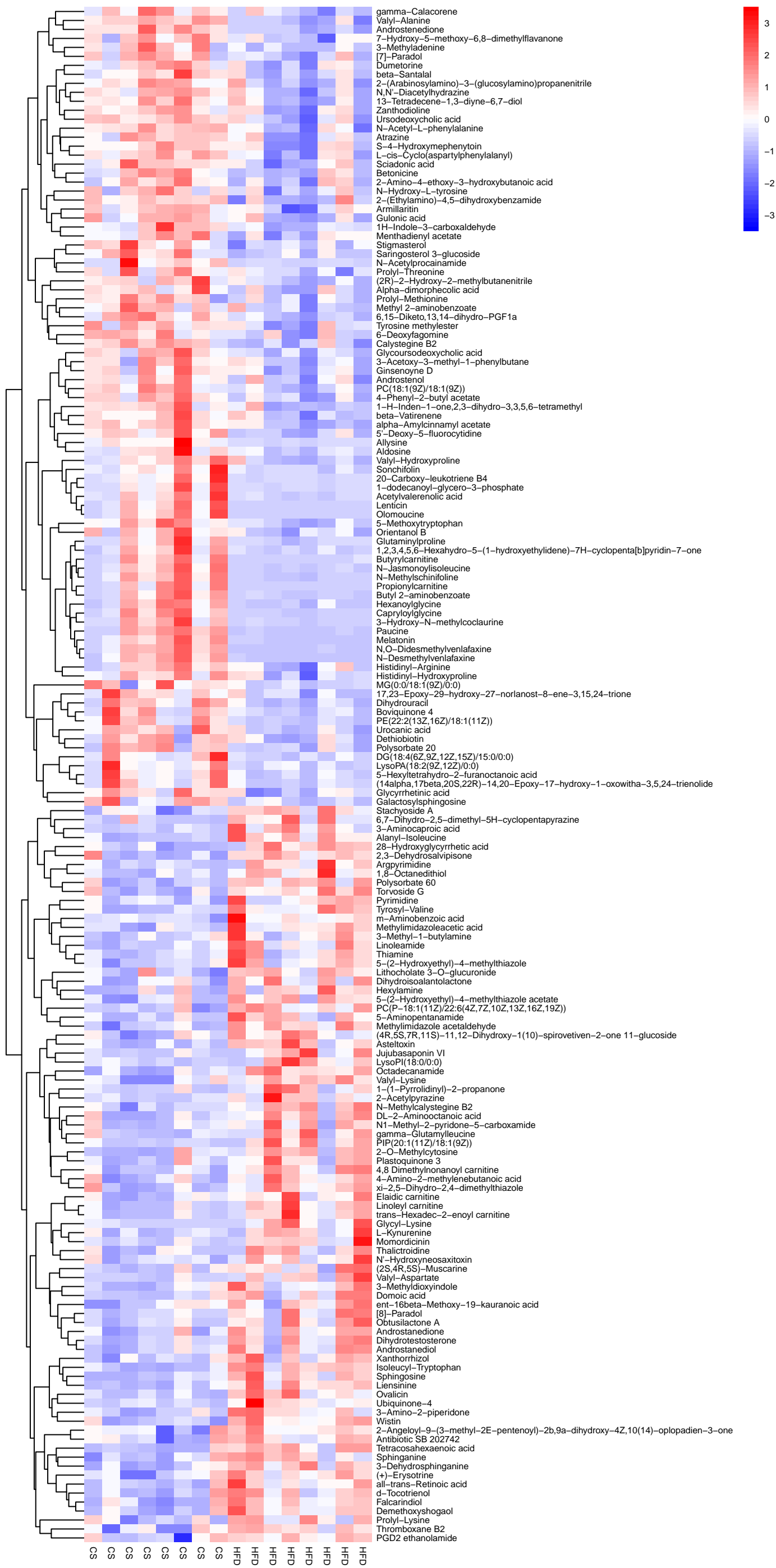

Supplement: Supplementary file 5 [file Data_Sheet_4.PDF]

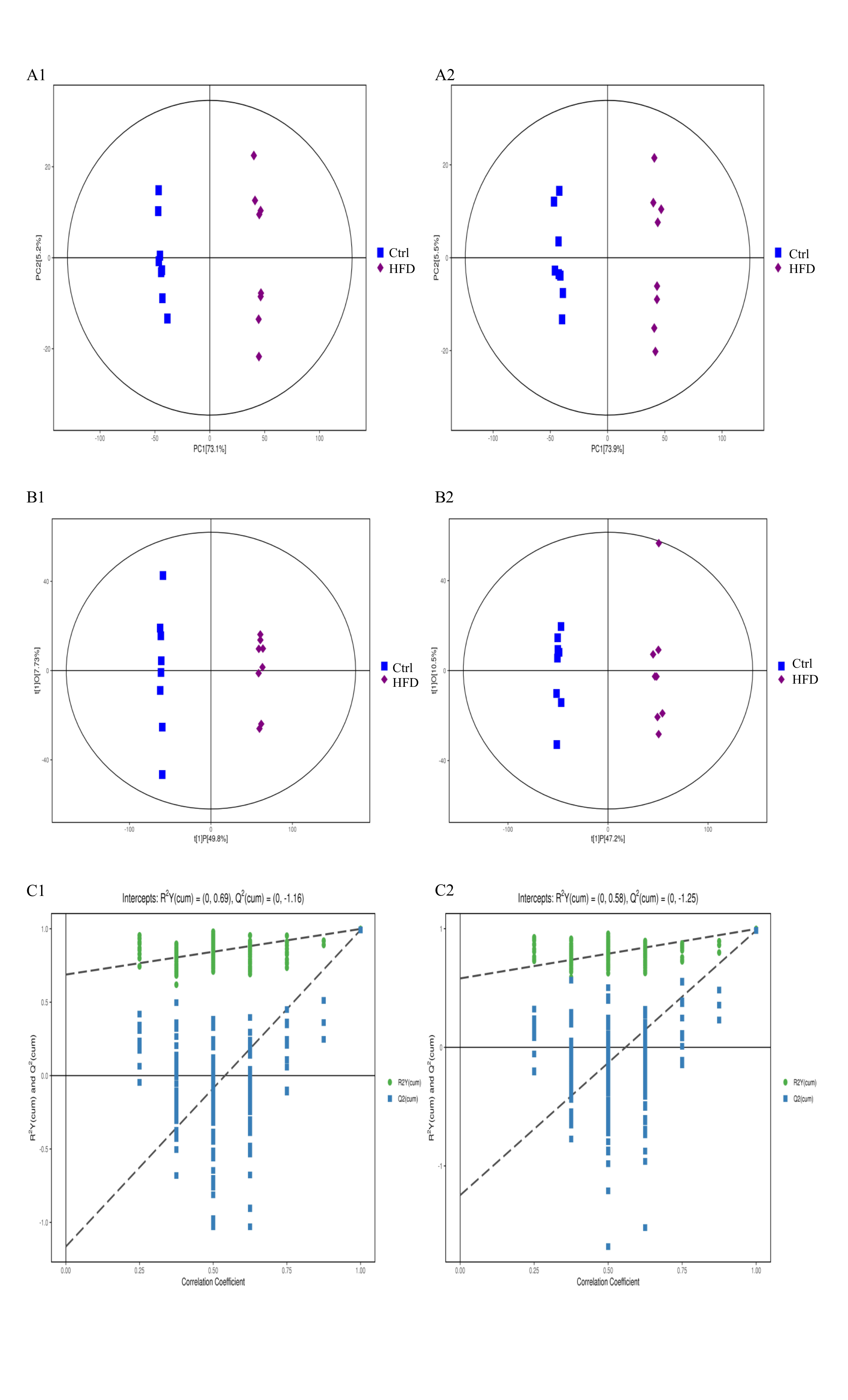

Supplement: Supplementary file 6 [file Image_1.TIF]

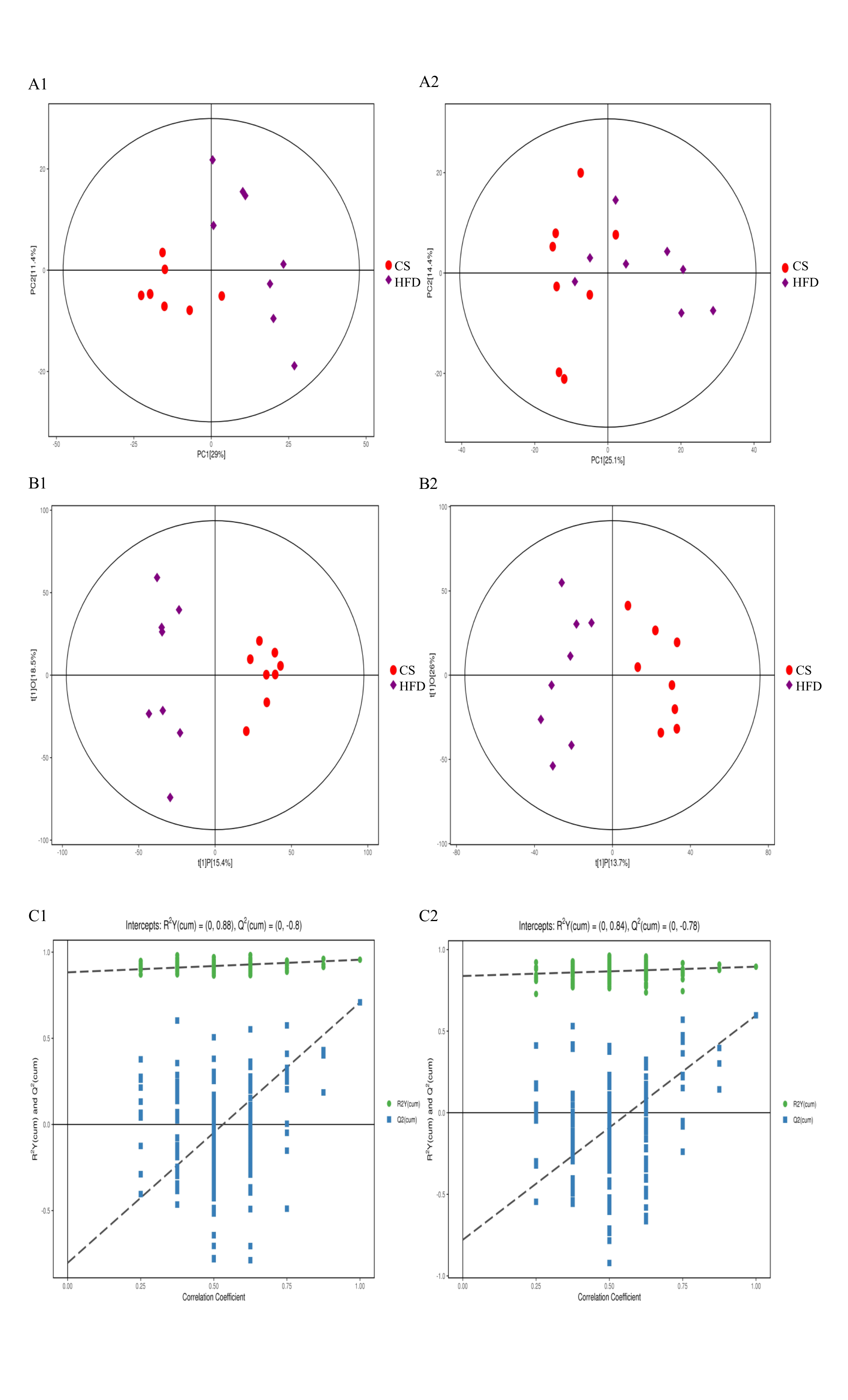

Supplement: Supplementary file 7 [file Image_2.TIF]

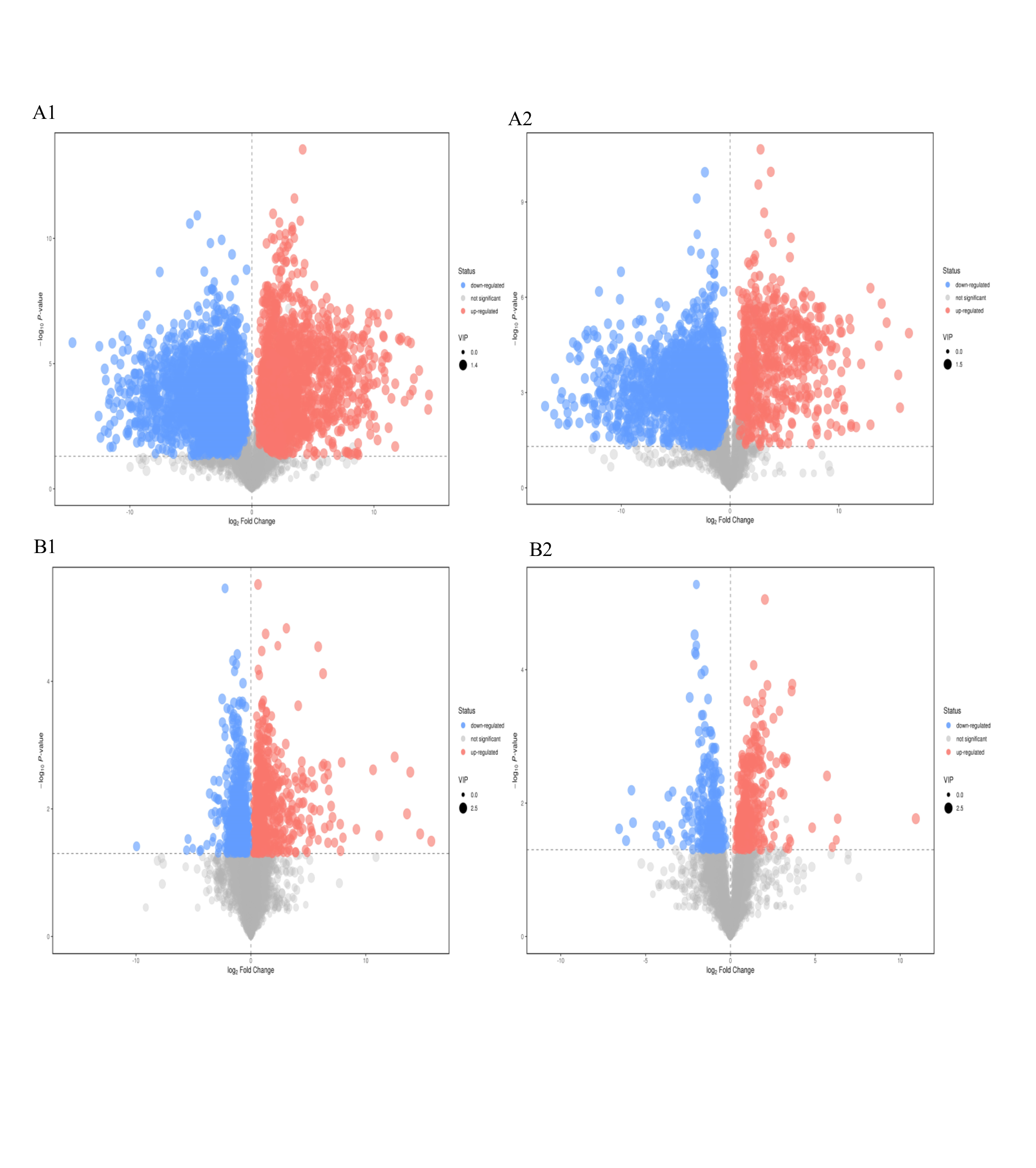

Supplement: Supplementary file 8 [file Image_3.TIF]
